# Screening the Molecular Framework Underlying Local Dendritic mRNA Translation

**DOI:** 10.3389/fnmol.2017.00045

**Published:** 2017-02-24

**Authors:** Sanjeev V. Namjoshi, Kimberly F. Raab-Graham

**Affiliations:** ^1^Center for Learning and Memory, The University of Texas at Austin, AustinTX, USA; ^2^Institute for Cellular and Molecular Biology, The University of Texas at Austin, AustinTX, USA; ^3^Department of Physiology and Pharmacology, Wake Forest Health Sciences, Medical Center Boulevard, Winston-SalemNC, USA

**Keywords:** mRNA, dendrites, translation, RNA sequencing, mass spectrometry, Kaede, synaptic tagging and capture hypothesis, synaptic plasticity

## Abstract

In the last decade, bioinformatic analyses of high-throughput proteomics and transcriptomics data have enabled researchers to gain insight into the molecular networks that may underlie lasting changes in synaptic efficacy. Development and utilization of these techniques have advanced the field of learning and memory significantly. It is now possible to move from the study of activity-dependent changes of a single protein to modeling entire network changes that require local protein synthesis. This data revolution has necessitated the development of alternative computational and statistical techniques to analyze and understand the patterns contained within. Thus, the focus of this review is to provide a synopsis of the journey and evolution toward big data techniques to address still unanswered questions regarding how synapses are modified to strengthen neuronal circuits. We first review the seminal studies that demonstrated the pivotal role played by local mRNA translation as the mechanism underlying the enhancement of enduring synaptic activity. In the interest of those who are new to the field, we provide a brief overview of molecular biology and biochemical techniques utilized for sample preparation to identify locally translated proteins using RNA sequencing and proteomics, as well as the computational approaches used to analyze these data. While many mRNAs have been identified, few have been shown to be locally synthesized. To this end, we review techniques currently being utilized to visualize new protein synthesis, a task that has proven to be the most difficult aspect of the field. Finally, we provide examples of future applications to test the physiological relevance of locally synthesized proteins identified by big data approaches.

## Introduction

Long-term memory formation relies on the modulation of synaptic efficacy – the strengthening or weakening of connections between a presynaptic and postsynaptic cell. Such changes are dependent on the alteration of the underlying neuronal architecture of the synapse through protein synthesis in the dendrites. In order for the changes made at the synapse to be long-lasting and consolidated, proteins must be synthesized rapidly in dendrites and spines. Thus, constitutive and activity regulated mRNA trafficking in neuronal cells allows localized protein synthesis in specific compartments or areas of the neuron far from the soma such as axons, dendrites, and spines (Jung and Holt, 2011). Consequently, many RNA transcripts coding for proteins that induce changes in synaptic efficacy are localized in dendrites and ready for rapid expression through local mRNA translation.

Historically, all mRNAs were thought to be exclusively translated in the soma. This dogmatic view was questioned when observations in a series of electron microscopy (EM) studies revealed the presence of polyribosomes in dendrites, specifically at the base of dendritic spines of the dentate gyrus (Bodian, 1965, 1972; Peters et al., 1976). It was not until 1983 that Steward and Levy provided the first quantitative evidence of synapse-associated polyribosome complexes and their localization (Steward and Levy, 1982). Steward and Levy (1982) hypothesized that synapse-associated polyribosome complexes may be necessary for the expression of proteins that constitute the synapse due to their proximity to dendritic spines. Their hypothesis was confirmed through numerous studies in the two decades that followed demonstrating the requirement for local protein synthesis in processes related to synaptic plasticity and learning. Some of these early studies demonstrated correlations between polyribosome numbers and synaptogenesis suggesting that the synapse-associated polyribosome complexes were the source of the proteins found in the postsynaptic density (PSD) (Steward and Falk, 1985, 1986; Palacios-Pru et al., 1988). This was followed by a number of key studies that identified select mRNA transcripts that were localized and translated in the dendritic spines (Steward et al., 1996; Steward and Schuman, 2001). Among these was the important discovery that BDNF-induced synaptic potentiation required local protein synthesis (Kang and Schuman, 1996).

Dendritic mRNA transport relies on complex formation of RNA granules. RNA granules contain RNA-binding proteins (RBPs) – which bind to sequestered mRNAs to inhibit their translation – as well as some translation factors, ribosomes, and other proteins that control translation (Kiebler and Bassell, 2006). Upon synaptic activation, select repressed mRNAs localized to the synapse are translated where the ribosome within the RNA granule can initiate rapid translation into the required protein product (Kim D. et al., 2013; Pimentel and Boccaccio, 2014). Notably, RBPs play a vital role in learning in memory. The absence of an RBP resulting from incorrect localization or dysfunction due to mutations may lead to aberrant translation or repression of specific mRNAs under its control resulting in a neurological disorder (Sephton and Yu, 2015). Furthermore, the activity of protein kinases, such as mammalian target of rapamycin (mTOR), is coupled to translation to facilitate processes related to learning and memory. Disruption of these processes can lead to neuronal dysfunction (Giese and Mizuno, 2013; Lipton and Sahin, 2014). Many animal disease models that reproduce both symptoms and genetic alterations seen in humans show dysregulated local mRNA translation (Pei and Hugon, 2008; Zang et al., 2009; Sharma et al., 2010; Ricciardi et al., 2011; Devi and Ohno, 2013; Ma et al., 2013).

In order to gain a more in depth view of the underpinnings of synaptic plasticity both in normal and diseased states many laboratories are initiating unbiased screens to identify (1) the mRNA transcripts localized to synaptic compartments, (2) the mRNA transcripts actively translated by the ribosome under specific cellular conditions, and (3) the protein kinases, RBPs, and microRNAs that control the timing and expression of locally translated mRNA. Importantly, researchers are combining classic techniques utilized since the 1950’s that have been extended and improved upon with highly specialized high-throughput methods to answer these questions and provide further insights into the molecular basis of neuronal function and neurological disease.

**FIGURE 1:**
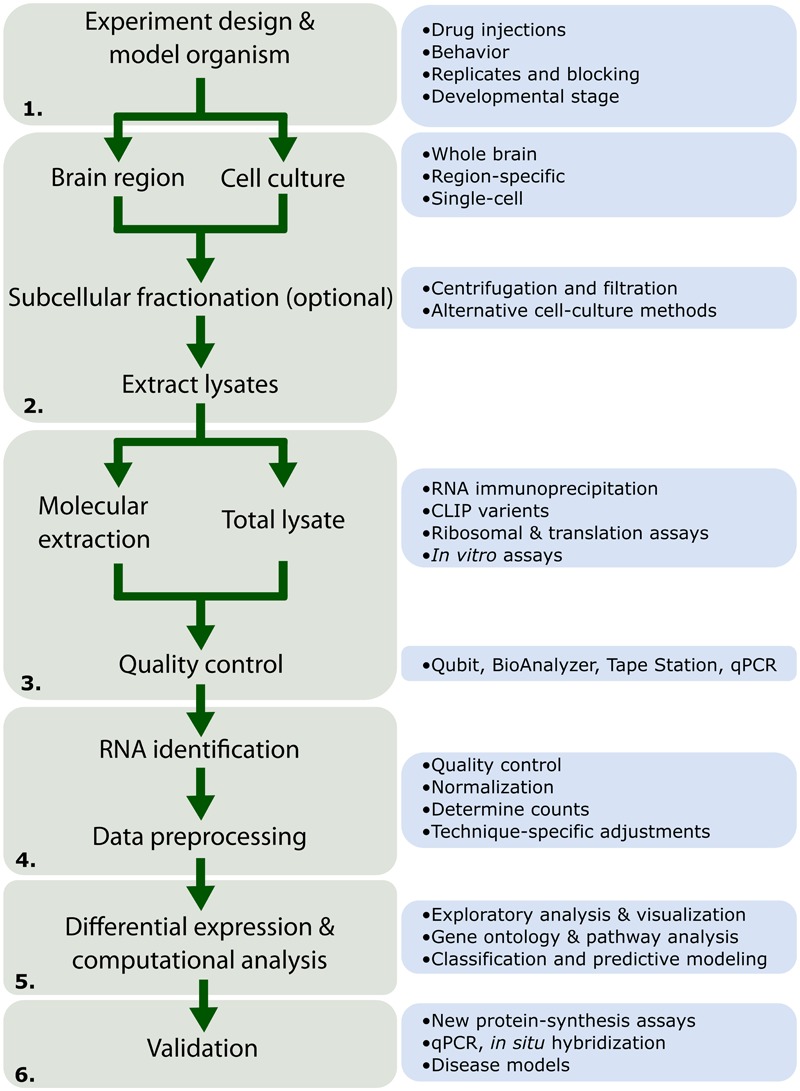
**Workflow for high-throughput RNA experiments.** Depiction of the different stages common to high-throughput RNA experiments as discussed in this review. (1) The experiment design stages should consider the scope of the project and must be designed with considerations for replicates and randomization. (2) High-throughput experiments may be performed *in vitro* (derived from cell culture). *In vivo* experiments may choose between the whole brain, subcellular fractions, or single-cells. Subcellular fractions can be obtained through a variety of different filtration methods as well as recent methods focusing on cell-cultures. This stage is especially crucial for studies in local translation as subcellular fractionation allows researchers to investigate spatial changes occurring in specific neuronal compartments. (3) RNA-Seq is typically performed on a total lysate population. Herein, we review a number of methods available for molecular extraction of RNA under various conditions (i.e., bound to proteins or the ribosome) as well as *in vitro* assays for assessment of RNA-binding properties. (4) After quality control assessment, the RNA may be sequenced. This is followed by a number of processing steps indicated in greater detail in **Figure 3**. (5) Computational analysis on RNA populations may reveal patterns and connections between processes previously unknown. Such experiments may also be followed up using protein identification techniques (detailed in **Table 3**). (6) Finally, validation can confirm novel findings seen in (5). Many new techniques exist allowing researchers to confirm both the spatial and temporal expression of numerous proteins and RNA systems involved in the control of synaptic plasticity through local translation.

Here we provide a historical overview and evolution of the major methods to identify and characterize locally synthesized proteins. These techniques have revealed the complex array of cell signaling and regulatory networks that govern local translation and synaptic plasticity in dendrites. We provide a general workflow for large-scale sequencing or proteomics projects highlighting general considerations and caveats at each stage (**Figure 1**). Then, we outline potential methods and strategies to validate findings of these large-scale projects in normal and disease rodent models.

## Planning the Experimental Design – General Considerations

Large-scale, high-throughput projects that analyze distributions of RNA and protein are generally costly and time-consuming. Notably, there is a tradeoff between replicates, depth of sequencing, and cost (Wang et al., 2011; Liu et al., 2013; Vijay et al., 2013). A flow chart outlining parameters to consider during the design phase are outlined in **Figure 1**. Beyond these parameters, randomization (Auer and Doerge, 2010; Cui, 2010; Fang and Cui, 2011; Williams et al., 2014) and replication are important. Tools such as Scotty^1^ have been created to aid in the determination of replicate number (Busby et al., 2013; Hart et al., 2013). Finally, performing a power analysis to determine the number of replicates for the experiment allows one to estimate of the effect size which in turn depends on the depth of sequencing.

## Rationale for Extracting Cell Specific and Subcellular RNA Populations

**FIGURE 2:**
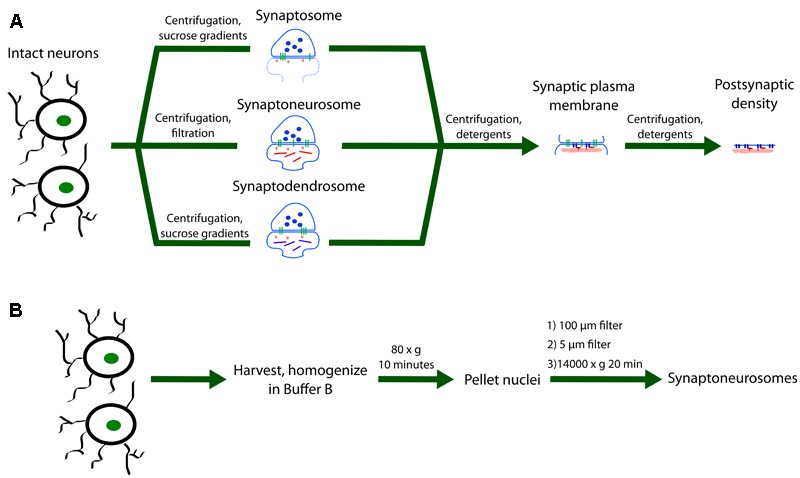
**Methodology for subcellular fractionation by centrifugation, filtration, and detergent application. (A)** Intact neurons are subjected to different combinations of centrifugation and gradient filtration. The synaptosome fraction (S) contains an enclosed presynaptic sack attached to a variable section of the postsynaptic membrane and its protein constituents. An alternative more popular preparation used in local translation studies is the synaptoneurosome fraction (SN). This fraction contains an enclosed sack on both the pre- and postsynaptic sides. The postsynaptic compartment has been shown to contain numerous components involved in synaptic plasticity processes such as local translation. The synaptodendrosome (SD) fraction is an alternative preparation to the SN fraction that has a slightly different chemical composition. Further application of centrifugation and detergents can produce the synaptic plasma membrane and the postsynaptic density (PSD). **(B)** SN preparation protocol modified from (Quinlan et al., 1999). Neurons are first harvested in Buffer B (20 mM HEPES, pH 7.4, 5 mM EDTA, pH 8.0) with added protease, phosphatase, and RNase inhibitors and homogenized. After pelleting at 80 × *g* for 10 min. the supernatant is filtered through a sterile 100 μm nylon filter followed by filtration through a 5 μm nylon filter. SN are then pelleted at 14,000 × *g* for 20 min. Pellets may then be solubilized in RIPA buffer (150 mM NaCl, 10 mM Tris, pH 7.4, 0.1% SDS, 1% Triton X-100, 1% deoxycholate, 5 mM EDTA, and added inhibitors) at 4°C overnight and centrifuged at 55,000 × *g* for 1 h. Alternative applications may require solubilization in a different buffer.

Approaches utilized to isolate synaptic mRNAs are vast. Biochemical isolation of synapses via centrifugation or filtration (see **Figure 2A**) and microdissection of dendritic fields in brain slices have provided a rich source of dendritic/synaptic mRNAs. More recently single-cell RNAseq has allowed researchers to classify cell transcriptome dynamics and determine cell-type diversity (Darmanis et al., 2015; Dueck et al., 2015, 2016). Data generated by these single-cell technologies offer promising opportunities for the field of learning and memory, especially when combined with data generated from RNA sequencing or proteomics of subcellular fractionations (i.e., the PSD as outlined in **Figure 2A**). These data, collectively, will provide powerful models guiding investigators to test translation of specific mRNAs in a cell and site-specific manner.

## Evolution of Biochemical Methods to Isolate Dendritic and Synaptic mRNAs or Proteins

### Utilizing Centrifugation, Filtration, and Density Sucrose Gradients to Isolate Dendrites

As early as 1956, researchers have been optimizing biochemical techniques to study synapses in isolation. Verity et al. (1980) were the first to show that the synaptosome (pre- and postsynaptic nerve endings) fraction contains polyribosomes, making it a promising candidate for the study of synaptic protein synthesis. The synaptosome (S) preparation combines centrifugation and sucrose gradient fractionation to create a cell fraction containing a sealed presynaptic structure attached to part of the postsynaptic membrane (Hebb and Smallman, 1956; Whittaker et al., 1964). The attached postsynaptic structure varies in size and may even contain the entire (unsealed) dendritic spine. However, the S preparation was seen to be insufficient for properly studying signal transduction and other events that took place in the postsynaptic cell. For this reason, the synaptoneurosome (SN) preparation was developed which includes both the sealed presynaptic structure and sealed postsynaptic compartment isolated through a series of filtration steps and low-speed centrifugation (Hollingsworth et al., 1985). This preparation is now commonly used to study and identify components of the postsynaptic membrane (**Figure 2B**).

Some of the earliest attempts to characterize proteins of the postsynaptic membranes required methods to further subfractionate the above synaptic fractions [including a slightly modified SN, referred to as synaptodendrosome (SD)] (Rao and Steward, 1991a, 1993). For example, the synaptic plasma membrane (SPM) subfraction, containing the proteins embedded in the plasma membrane around the synapse, can be purified from the synaptic fractions (Blackstone et al., 1992; Bermejo et al., 2014). The SPM fraction can be further fractionated to obtain the PSD subfraction, the large scaffolded complex of proteins found clustered at the edge of the postsynaptic membrane (Carlin et al., 1980; Villasana et al., 2006). Rao and Steward (1991a) isolated SPMs by subjecting S to further gradient fractionation combined with the detergent Triton X-100 and demonstrated that protein synthesis was occurring in this region. Expanding on this early finding by Rao and Steward (1991a); Niere et al. (2016) subjected the PSD fraction, isolated in a similar manner, to mass spectrometry to show that 75% of the PSD changes in composition upon inhibiting the protein synthesis pathway mTOR for only 1 h *in vivo*. Thus, combining classic biochemical subcellular techniques with big data approaches has vastly expanded our knowledge of how dynamic protein synthesis occurs in the PSD.

### Limitations to Biochemical Synapse Isolation and Potential Strategic Measures Utilized to Overcome These Limitations

Though subcellular fractionation is still commonly employed to study molecular events at the postsynapse, there are limitations. The S, SN, and SD preparations are considered impure since they cannot successfully remove glial fragments (Chicurel et al., 1990; Rao and Steward, 1991b, 1993). Hollingsworth et al. (1985) noted “unidentifiable debris” when they analyzed the preparation by EM which suggests that there may be foreign protein and RNA carryover from membrane fragments or other fractions not normally associated with axons or dendrites. An additional concern is the presence of somatic contaminants. EM provides the most rigorous check, however, one might quickly screen for somatic contamination by examining a sample of the synaptic preparation versus the total homogenate with a nuclear stain such as DAPI or western blotting for a nuclear protein such as NeuN (Sosanya et al., 2013). Moreover, after large scale screens one may subtract possible contaminants bioinfomatically. To determine axonal or dendritically expressed transcripts, Schuman and colleagues sequenced RNA isolated from the stratum radiatum and lacunosum moleculare of the rat hippocampus and subtracted transcripts enriched in glia, interneurons, nucleus, mitochondria, and blood vessels based on cell type-specific transcriptome data from previous publications and online databases (Cajigas et al., 2012). While this technique may eliminate candidates that are expressed both in glia and neurons, it does allow one to follow up putative dendritic mRNAs with more certainty. In spite of these limitations, these preparations have provided the basis for many studies that have moved the field forward.

### Approaches to Isolating Dendrites

Eberwine et al. (2001) were the first to isolate dendrites from neuronal cell bodies in hippocampal cell cultures. A micropipette was used to microdissect the dendrite in which RNA was isolated. Differential display and microarray analysis of mRNA isolated in this manner, provided the first large-scale analyses of the mRNA present in the dendrite (Miyashiro et al., 1994; Eberwine et al., 2001). Considering the limitations of these early assays, remarkably the authors estimated that ∼400 mRNAs reside in the dendrites (Eberwine et al., 2001).

Since then, less labor intensive methods have been developed. One clever technique capitalized on the fact that neuronal and glial cell bodies are typically at least 10 μm in size. By plating hippocampal neurons onto PET membranes with 3 μm pores neuronal processes are separated from neuronal cell bodies and glia. Thus, neuronal processes can be isolated by scrapping the bottom of the filter (Torre and Steward, 1992; Poon et al., 2006). Additionally, laser capture microdissection of neurites has also been employed successfully to catalog the mRNAs present in dendrites (Kye et al., 2007). More recently, Lovatt et al. (2015) have refined this method to isolate a single neuron from cell culture including dendrites. These techniques offer promising alternatives to complement the standard subcellular fractionation methodologies. Through mRNA amplification technologies, it is now possible to perform cell and compartment-specific identification of synaptic mRNAs.

## What Isolating Regulatory Factors Can Tell You About Synaptic Efficacy

### Isolation of RNA-Binding Proteins and RNA Populations

RNA-binding proteins and many types of RNAs play a vital role in learning and memory by controlling transcript localization and availability in dendrites (Sephton et al., 2011; Aksoy-Aksel et al., 2014; Lenzken et al., 2014; Smalheiser, 2014; Zhou et al., 2014). In the past decade, a number of important high-throughput techniques have been developed concurrently with specialized deep sequencing technology that has allowed researchers to elucidate the RNA populations bound to RBPs or ribosomes on an unprecedented scale. In the following sections, we will outline the basic principles of these techniques and compare their advantages and disadvantages (**Table 1**). We will also consider a number of *in vitro* selection-based techniques that complement *in vivo* assays.

**Table 1:** Summary of methods for identifying RNA–protein interactions.

Assay type	Technique	Advantages	Disadvantages	Reference
Immunoprecipitation	RIP-SEQ/RIP-CHIP	Recover full length-RNA	High background, antibody-based	Keene et al., 2006; Oeffinger et al., 2007; Jain et al., 2011; Dahm et al., 2012
RNA-Protein interactions through CLIP-SEQ	HITS-CLIP	High resolution	Difficult, low cross-linking efficiency, cross-linking artifacts, antibody-based, RT-PCR mispriming, cannot distinguish between single protein binding and protein complex binding	Licatalosi et al., 2008; Darnell, 2010; Kishore et al., 2011; Zhang and Darnell, 2011; Gillen et al., 2016
	PAR-CLIP	Very high resolution, high cross-linking efficiency	Difficult, expensive, 4-SU toxic, high background, antibody-based, low alignment %	Hafner et al., 2010; Spitzer et al., 2014
	iCLIP/iCLAP	Very high resolution, RT-PCR does not stall at crosslink site	Very difficult to perform	Konig et al., 2010; Wang Z. et al., 2010; Sugimoto et al., 2012; Huppertz et al., 2014
	CRAC	Affinity purification-based, less background	Difficult, tag may interfere with protein function	Granneman et al., 2009; Bohnsack et al., 2012
	PIP-SEQ	Does not us UV cross-linking, identifies non-Poly(A) transcripts	Difficult, very new method	Silverman et al., 2014
RNA Structure	CLASH-SEQ	Identification of RNA–RNA duplexes	Use of two adaptors results in ambiguity, ligation reaction inefficient	Kudla et al., 2011
	HiCLIP	Improves upon CLASH-SEQ, can identify long RNAs	Difficult, very new method	Sugimoto et al., 2015
Ribosome-based	RIBO-SEQ	Greatly improves on past footprinting techniques, high-throughput	Lysis preparation may change ribosomal distribution, stalled ribosome may bias results	Esposito et al., 2010; Masek et al., 2011; Gandin et al., 2014
	TRAP-SEQ	Easier to perform than RIBO-SEQ	Lacks RIBO-SEQ specificity	Jiao and Meyerowitz, 2010
*In vitro* binding	SELEX	Quick, easier to perform than alternative *in vivo* methods	High affinity motif bias, identifies non-physiological interactions	Darmostuk et al., 2015
	RNAcompete	Quick, easier to perform than alternative *in vivo* methods	RNA secondary structures may affect binding assay, identifies non-physiological interactions	Ray et al., 2009
	SEQRS	Identifies many more motifs than RNAcompete	Identifies non-physiological interactions	Campbell et al., 2012
	RNA Bind-n-Seq	Greatly improves on other *in vitro* methods, compliments CLIP-based assays	Identifies non-physiological interactions	Lambert et al., 2014

#### RNA Immunoprecipitation Sequencing/Microarray (RIP-SEQ/RIP-CHIP)

The RNA immunoprecipitation (RIP) has been used previously to identify targets of RBPs involved in neurological dysfunction (Buckanovich and Darnell, 1997; Napoli et al., 2008; van der Brug et al., 2008; Fernandez et al., 2015). High-throughput RIP-SEQ serves as a useful tool in determining RNA populations bound to proteins involved in local translation. RIP, similar to the protein-based immunoprecipitation procedure, has been optimized in order to preserve the RNA-protein complex during the lysing step such as gentle-freeze thawing (Keene et al., 2006; Oeffinger et al., 2007; Jain et al., 2011; Dahm et al., 2012). Other modifications in RIP protocols are to ensure that free RNAs released during lysis do not bind non-specifically to the beads or the RBP, a phenomenon that has been observed previously and contributes to the high background of some RIP experiments (Mili and Steitz, 2004). Following some of the procedures outlined by Jain and colleagues, it is possible to achieve minimal or negligible levels of background binding (Jain et al., 2011). While crosslinking with formaldehyde to bind the protein–protein or protein–RNA structures together may help in ensuring complex isolation, in some cases it may not lower the level of background binding (Penalva et al., 2004). Finally, like many other high-throughput techniques, there are limitations including epitope accessibility to the antibody, as well non-specific binding inherent with antibody-based procedures. Importantly, RIP is not able to reveal precise binding sites like other crosslinking techniques discussed below; however, it can reveal the full-length transcript of RNAs bound to the protein of interest *in vivo*.

#### Crosslinking-Based Techniques to Identify RBP-Bound RNAs

##### HITS-CLIP

Cross-linking and immunoprecipitation (CLIP) was originally developed by the Darnell Lab to study interactions between RBPs and its target RNAs (Ule et al., 2003, 2005). When combined with high-throughput sequencing, the modified protocol is referred to as HITS-CLIP (Licatalosi et al., 2008; Darnell, 2010). The HITS-CLIP technique allows researchers to perform mapping of RBP binding sites on RNA in a high-throughput manner. Modifications have also been made to the HITS-CLIP procedure that now allow up to single-nucleotide resolution of RBP binding sites (Kishore et al., 2011; Zhang and Darnell, 2011). While HITS-CLIP allows for fine resolution of RNA-protein interaction sites, the crosslinking procedure can introduce artifacts and during reverse transcription mispriming events can occur (Kishore et al., 2011). Notably, recent improvements have been made to the procedure to minimize mispriming artifacts through the use of two specialized primers during the reverse transcription step (Gillen et al., 2016). One of the biggest limitations to HITS-CLIP is the low cross-linking efficiency, which has been reported to be ∼5% (Darnell, 2010). Still, HITS-CLIP and its derivatives have been utilized to identify RBP binding sites for proteins involved in local translation, to determine microRNA bindings sites, and identify RNA targets for proteins involved in neurological and developmental dysfunction (van der Brug et al., 2008; Darnell et al., 2011; Ascano et al., 2012a; Ince-Dunn et al., 2012; Lagier-Tourenne et al., 2012; Wagnon et al., 2012; Boudreau et al., 2014; Weyn-Vanhentenryck et al., 2014; Scheckel et al., 2016).

##### PAR-CLIP and iPAR-CLIP

Photoactivatable-ribonucleoside-enhanced crosslinking and immunoprecipitation (PAR-CLIP) was introduced in 2010 to address the issue of low crosslinking efficiency in HITS-CLIP and background RNA in samples from non-crosslinked proteins (Hafner et al., 2010; Spitzer et al., 2014). A photoreactive nucleoside analog of uridine (4-SU) and guanosine (6-SG) are added to cultured cells which increases crosslinking efficiency. 4-SU causes a thymidine to cytidine transition during the reverse transcriptase reaction thus indicating the exact crosslink sites. While an improvement over HITS-CLIP there are still limitations to this technique. First, 4-SU is believed to be toxic to cells at concentrations used in PAR-CLIP by inhibiting processing of 47S rRNA, thus affecting the experimental results (Burger et al., 2013). Second, PAR-CLIP was quantitatively shown to have reproducible levels of background signals, necessitating empirical determination of background as an extra step in the analysis (Friedersdorf and Keene, 2014). Finally, PAR-CLIP also suffers from poor alignment issues, as aligned reads can be as low as 20% of total reads after RNA sequencing (Hafner et al., 2012). Recently, PAR-CLIP has been adapted to for *in vivo* identification of RBP mRNA targets, called iPAR-CLIP, with the “i” standing for *in vivo*, and has been shown to be less toxic overall (Jungkamp et al., 2011). Importantly, PAR-CLIP has been used to understand the binding of RBPs whose dysregulation has been shown to play a role in neuronal diseases. Some example include Rbfox3 which was found to have a unique function in the regulation of pri-mRNA (Kim et al., 2014), the first identification of two FMRP binding motif sequences (Ascano et al., 2012b), and the first report to identify all the targets of the FET protein family (FUS, EWSR1, and TAF15) (Hoell et al., 2011). Thus, PAR-CLIP has moved the field forward by determining binding motifs/targets for RBPs allowing for the investigators to answer questions on how RBPs contribute to coordinated translation with synaptic plasticity.

##### iCLIP, iCLAP, and CRAC

Individual-nucleotide resolution CLIP (iCLIP) was developed in response to the data showing that the reverse transcription reactions truncate at the crosslink sites in HITS-CLIP and PAR-CLIP (Konig et al., 2010; Sugimoto et al., 2012; Huppertz et al., 2014). The iCLIP method adds a circular PCR amplification step that allows researchers to determine the sequence of cDNAs that would normally be truncated in other CLIP methods. During cDNA synthesis, truncation will occur at the crosslink site. The cDNA is then circularized, linearized, and PCR amplified to determine the region of the protein binding site at the crosslink. While iCLIP does resolve some of the issues that other CLIP methods face, it is technically challenging and has extra steps that could compromise RNA stability, which is already limiting. Additionally, the extra manipulation at the PCR amplification stage could bias the final results. iCLIP has another variation known as individual-nucleotide resolution crosslinking and affinity purification (iCLAP) which uses a two-step affinity purification. This technique may be an option if antibodies are not available and may lower background (Wang Z. et al., 2010). Finally, another technique that uses affinity purification is cross-linking and analysis of cDNAs (CRAC). This technique requires RBPs to be tagged with protein A and hexahistidine sites for IgG purification followed by nickel-affinity purification (Granneman et al., 2009; Bohnsack et al., 2012). This technique has been used to uncover spliceosomal RNA–protein interactions and may prove to be a useful method for cleanly isolating an RBP within a complex. Thus, each technique has been optimized to overcome specific limitations, to provide researchers a tool kit to address their specific question (**Table 1**).

##### PIP-SEQ, HiCLIP, and CLASH-SEQ

Protein interaction profile sequencing (PIP-SEQ) is another more recent high-throughput method that can map RNA-protein interactions in an unbiased, transcriptome-wide manner, rather than selectively with specific RBPs (Silverman et al., 2014). Importantly, this method provides information on possible secondary structures within the mRNA. Notably, RNA secondary structures have already been observed as a control mechanism in long-term memory formation as well as a feature of 3′UTR recognition sequences for localization to the dendrite (Martin and Ephrussi, 2009). HiCLIP and CLASH-SEQ are two related methods that can be used to map RNA secondary structures (Kudla et al., 2011). CLASH-SEQ is a high-throughput method that allows for the transcriptome-wide level identification of secondary structures via analysis of RNA duplexes. HiCLIP improves upon the biases and limitations of CLASH-SEQ by adding another adapter that allows identification of RNA–RNA duplexes with greater precision (Sugimoto et al., 2015). Collectively, these techniques can provide answers to long-sought after questions regarding how secondary structure may encode dendritic targeting and translational regulation signals that investigators have struggled with for several years.

##### RNA interactome capture

Another crosslinking method that differs from the CLIP-based methods has recently been developed. RNA interactome capture can be used to survey the full repertoire of both protein and RNA interacting physiologically within cells (Castello et al., 2013, 2016). UV irradiation is used to crosslink RBPs to polyadenylated RNAs which are then isolated using oligo(dT) magnetic beads. Next, RNA and protein are separated and analyzed by RNAseq and mass spectrometry respectively. Like all crosslinking methods, it is limited by crosslinking efficiency. Furthermore, it will not be able to isolate RBPs bound to non-polyadenylated RNA. Thus, for the first time, investigators can isolate Protein–RNA interactions as a network, providing insight into how RBPs work in concert to regulate mRNA translation of plasticity related proteins.

#### Ribosomal/Translation-Based Methods

##### RIBO-SEQ/ARTSEQ and Polysome Profiling

The analysis of global mRNA levels within a cell population is commonly used to measure gene expression. However, this may not be a sufficient metric as mRNA levels do not necessarily correlate to protein expression levels due to an extra layer of translational control at the level of the ribosome. Therefore, ribosome-specific RNA methods have been developed to better understand the dynamics and control of mRNA translation. Translation serves as a rapid mechanism by which the cell can finely control the amount of protein to be expressed from a particular mRNA in both the spatial and temporal dimensions. Such regulation of translation serves a major function in both memory formation and synaptic plasticity thus necessitating the need for methods able to profile mRNAs under active translation (Costa-Mattioli et al., 2009; Buffington et al., 2014). One approach to identifying mRNAs under active translation is polysome-profiling in which ribosomes with high translation efficiency are selectively isolated by polysome gradient fractionation, followed by RNA isolation and high-throughput sequencing or microarray (Esposito et al., 2010; Masek et al., 2011; Gandin et al., 2014). More recently, the development of ribosome profiling sequencing (RIBO-SEQ or active mRNA translation sequencing, ARTSEQ) has provided a genome-wide approach used to identify mRNA being actively translated by the ribosome without consideration of translational efficiency. In this context, translational efficiency is defined as the mean ribosomal footprint counts for a given mRNA, a quantitative measure of the degree of ribosomal occupancy (Ingolia et al., 2009; Ingolia et al., 2012). All ribosomes in active translation are isolated and the associated untranslated mRNA is then removed and digested. Then, the rRNA is depleted from the samples and the actively translated mRNA is reverse transcribed and sequenced. The fragments of RNA protected from digestion are then mapped to a reference genome thereby providing the location of the ribosome on various mRNA at a nucleotide-scale. Since ribosome profiling uses a footprinting approach, it is able to reveal the precise binding sites of the ribosomes across the mRNA and provide quantitative measures of expression. These features are not possible with the traditional polysome profiling approach. Due the high sensitivity of RNA sequencing approaches, these methods can provide detailed information about mRNAs undergoing translation, thus allowing researchers to better understand how the synapse is actively changing in the context of learning and memory.

##### TRAP/TRAP-SEQ and RiboTag

Translating ribosome affinity purification (TRAP) is another method for mapping actively translated mRNAs using EGFP-tagged ribosomal protein L10 (RPL10). The technique is performed in transgenic mice containing Bacterial Artificial Chromosomes (BACs) (Heiman et al., 2008). The technique was later extended for use in RNAseq in a method known as TRAP sequencing (TRAP-SEQ) which uses His and FLAG epitope-tagged ribosomal protein L18 (RPL18) to immunopurify translating ribosomes (Jiao and Meyerowitz, 2010). However, because this assay captures whole ribosomes (both polysomes and monosomes) the translational state of the mRNA of interest will not be as high-resolution as that obtained from ribosomal profiling or polysome profiling. A recent study has attempted to modify the TRAP-SEQ method in such a way that it is possible to extract ribosome-bound mRNA specifically from dendrites (Ainsley et al., 2014). RiboTag is another recently developed method for a mouse transgenic line in which the ribosomal protein L22 (RPL22) gene has been HA tagged before the stop codon. This mouse can then be crossed with a mouse line containing cell type-specific Cre-recombinase thus creating HA-tagged ribosomes in the cell-type of choice. Immunoprecipitation will recover ribosome-bound mRNA in the chosen cell type (Sanz et al., 2009). This method circumvents the need for the BAC required in TRAP-SEQ. Similar to the HA-tagging and TRAP technology described above, another method has recently been developed that allows ribosomes to be GFP-tagged, but only immunoprecipitated from cells that project to a specified brain region (Ekstrand et al., 2014). Thus, TRAP-SEQ provides extensive information about mRNA populations undergoing translation and with the modification of circuit specific GFP-tagged ribosome it’s now possible to examine coordinated mRNA translation in between specific brain regions.

#### *In vitro* Binding Assays

##### RNA-SELEX, RNAcompete, SEQRS, and RNA Bind-n-Seq

*In vitro* binding assays provide a means for surveying the RNA-binding preference of RBPs. While some of the results may be non-physiological, they are useful for motif identification and can complement other antibody-based methods to separate falsely identified RNA targets. Systematic evolution of Ligands by EXponentional Enrichment (SELEX) was developed in the 1990s as a way of assessing the binding affinity of proteins to a pool of random oligonucleotides. The oligonucleotide library is incubated with the target protein of interest. Candidate oligonucleotides that bind the protein are reverse transcribed, amplified, and used to seed a new round of selection with the protein of interest. After several rounds, the RNAs are sequenced. These RNA molecules represent sequences with high affinity to the protein of interest. SELEX is useful for determining novel RNA–protein interactions and RBP motif discovery. Many improvements have been made to SELEX over the years and RNA SELEX now exists in a high-throughput form, giving researchers the ability to assess possible RNA sequences that can bind to a given molecular target (Darmostuk et al., 2015). Other attempts to assess RBP binding preference include RNAcompete (Ray et al., 2009). Here, a custom-made microarray is used to produce a pool of RNAs (29–38 nucleotides in length) which are either unstructured or contain stem-loops. These RNA molecules are then made double-stranded through primer extension on the array. After release from the array, GST-tagged RBPs are incubated with the RNA pool. The RNA bound to the RBP is then removed, extracted, labeled, and hybridized to a microarray for high-throughput analysis. SEQRS is another method that builds upon older forms of *in vitro* selection such as RNA SELEX. DNA oligonucleotides with a random 20 nucleotide sequence are transcribed to RNA and then incubated with a recombinant protein of interest. The RNAs are then extracted, converted to cDNA, and sequenced (Campbell et al., 2012). RNA Bind-n-Seq was recently developed with an aim to simplify the method and avoid the bias inherent to CLIP (Lambert et al., 2014). The method uses a random pool of RNAs which are incubated with a purified RBP present at different concentrations. The RBPs are pulled-down using streptavidin magnetic beads, the RNA is extracted, converted to cDNA, and sequenced. The authors suggest that this method be used in tandem with CLIP-based techniques to filter out false positives.

## High-Throughput Assays

For RNA samples, the newest technology available is RNAseq. In the last decade, RNAseq has replaced microarray technology primarily because it is believed to be more accurate, more sensitive, and has a broader dynamic range (Marioni et al., 2008; Fu et al., 2009; Zhao et al., 2014). However, the analysis pipelines are not completely standardized, as they are for microarrays. RNAseq pipelines also require in-depth bioinformatics analysis. Like microarrays, mass spectrometry is an older technique with a more standard analysis pipeline that has been reviewed in detail elsewhere (Slonim and Yanai, 2009; Lavallée-Adam et al., 2015). Here we provide a step by step guide to RNAseq technology and approaches to analysis downstream.

### RNA-Sequencing (RNAseq)

**FIGURE 3:**
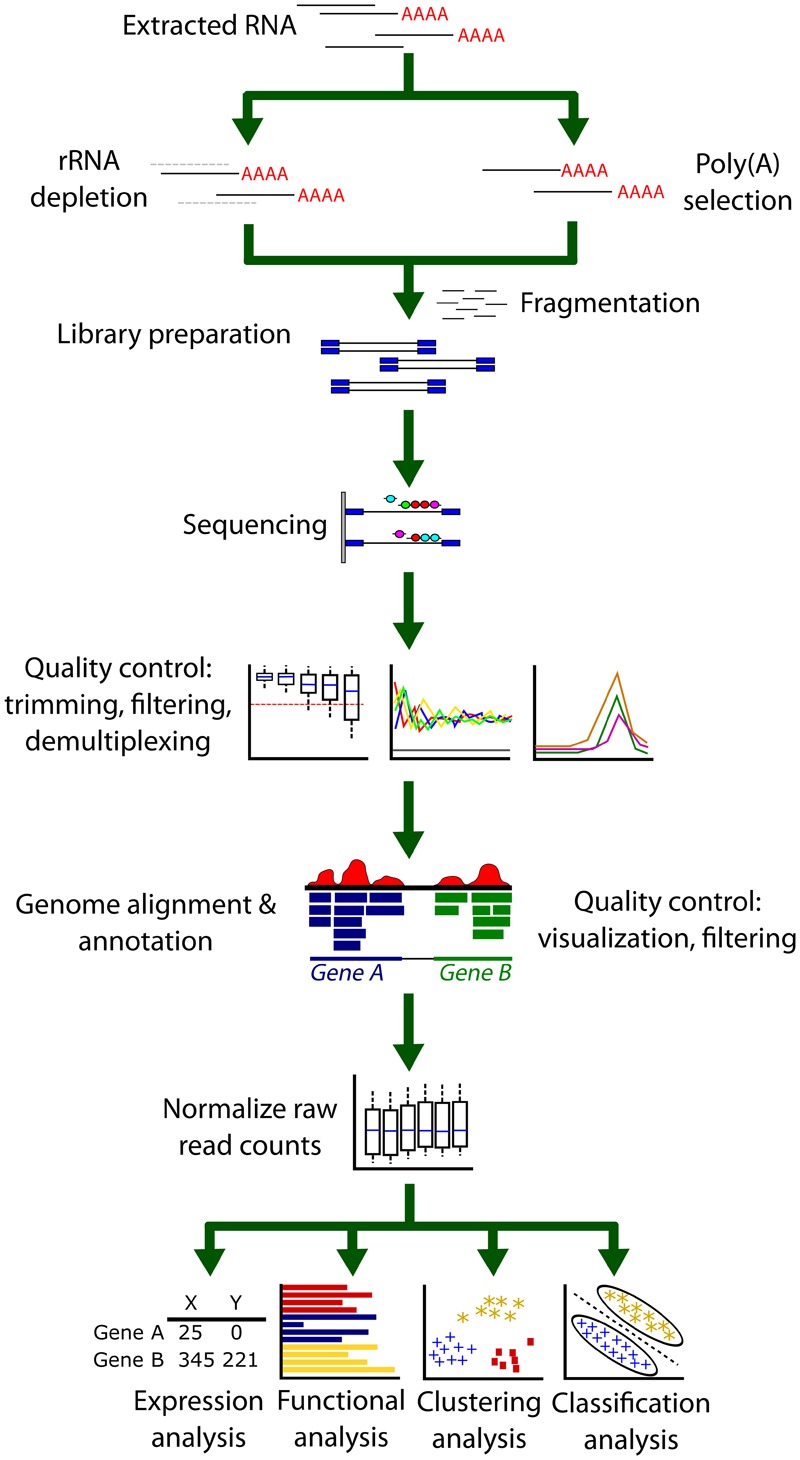
**A typical RNA-Seq analysis pipeline.** Extracted RNA first undergoes either rRNA depletion or Poly(A) selection to remove ribosomal RNA contamination as this represents a significant portion of RNA extracted from cells. A cDNA library is then prepared from this RNA in a process that may include a PCR amplification step. After sequencing, the output FASTQ files are then inspected to confirm the success and quality of the sequencing reaction. Trimming of low-quality base calls or filtering poor quality reads may be necessary. Next, FASTQ files are aligned to a genome or transcriptome using an aligner. Many aligners also simultaneously annotate the alignment files. It is good practice to determine the success of the alignment by using a genome browser as well as other quality control checks for alignment percentage and remaining rRNA contamination. The resulting output files (usually BAM or SAM format) must now be counted using a read counter. These raw reads (or sometimes the BAM/SAM alignment files) are then passed to software or algorithms that can perform further analysis. Between or within sample comparisons of counts cannot be performed until the library counts have been normalized.

Next-generation sequencing technology (NGS) represents the latest technologies used in high-throughput sequencing. A number of different platforms are available including Illumina, Ion Torrent (Fischer), Roche 454 (Roche), and SOLiD (Life Technologies). Various approaches to sequencing have been developed and improvements have been made over the years (Goodwin et al., 2016). Here we will focus on the solid-phase bridge amplification technology pioneered by Illumina. The Illumina platform is currently the most widely used and the company’s HiSeq 2000 boasts the lowest sequencing cost per gigabyte of data compared to other platforms and a low error rate (Loman et al., 2012; Quail et al., 2012). However, for greater sensitivity and a lower error rate (which may be of use in SNP analysis, for example), the SOLID platform (Applied Biosciences) may be preferred. An overview of a typical RNAseq pipeline is summarized in **Figure 3**.

#### Quality Control

Before sequencing begins, RNA obtained from cell samples are used to generate a cDNA library (Head et al., 2014). A common pitfall is degraded or impure RNA. Low quality RNA generates noisy data whose sequences can be difficult to reconstruct during the data preparation stages. Furthermore, sequencing degraded RNA leads to high variability and can impact interpretation and differential gene expression (DGE) analysis leading to the possibility of overfitting in classification analysis. The RNA integrity number (RIN, ranging from 1 to 10) is a statistical measure of RNA integrity that has been developed to assess RNA quality in a particular sample (Schroeder et al., 2006). Typically, a RIN of 7–10 is recommended for library construction unless the RNA was derived from rare tissue and obtaining higher quality RNA is costly or impossible. However, it appears possible to construct libraries and analyze data from moderately degraded RNA (RIN = 4–6) using appropriate statistical corrections (Gallego Romero et al., 2014; Sigurgeirsson et al., 2014; Cieslik et al., 2015). As an alternative to using RIN, qPCR may also be used as a quality assessment if potential transcripts are already known (Vermeulen et al., 2011). For RNA-protein interaction studies, qPCR can be used to assess the validity of target samples by using primers for known RNA targets of the immunoprecipitated RNP. This method does not assess the entire extracted RNA sample but can be a useful tool to demonstrate the success or relative quality of the RNA isolation technique.

#### rRNA Removal

After RNA quality control (QC), the samples typically undergo a selection stage for the removal of ribosomal RNA (rRNA). Since RNAseq will provide sequences for the most abundant RNAs in the sample, rRNAs must be removed since they comprise >90% of the RNA isolated from a cell population. There are two major strategies to approach this issue and recent work suggests that both methods introduce biases in sequence coverage (Lahens et al., 2014). However, both of these methods are currently in wide use and since no alternatives exist, efforts are ongoing to decrease bias (van Dijk et al., 2014). Since mature, processed mRNAs contain a poly(A) sequence, oligo(dT) beads can be used to select for only mature mRNAs in a technique called poly(A) selection, effectively removing the rRNA from the sample. While cheaper and of higher sensitivity compared to the alternative choice, the ribosomal RNA (rRNA) depletion technique, it will not pick out non-coding RNAs and may detect less genes overall (Cui et al., 2010; Zhao W. et al., 2014). There are still concerns of 3′ end bias for poly(A) selection sequences but recent studies suggest that this may be overcome with appropriate statistical corrections (Roberts et al., 2011). rRNA depletion utilizes beads consisting of sequences complimentary to rRNA. Currently, this method is more expensive and does not remove all rRNA (Cui et al., 2010). However, it is necessary for sequencing non-coding RNA.

#### High Throughput Sequencing

Following rRNA depletion, the RNA is sheared into small fragments and converted to cDNA by reverse transcription. Unique DNA linkers are ligated onto the 3′ and 5′ ends of the isolated oligonucleotide. Special capture sites are then added on to the 3′ and 5′ ends of the molecule which allows them to be anchored onto a solid support surface during the sequencing steps. PCR amplification is often performed at this stage depending on the amount of RNA available. RNA sequencing is often multiplexed so multiple experiments can be run simultaneously. This is accomplished by attaching a barcode between the 3′ linker and the 3′ end capture site. These unique barcodes can be used to differentiate between multiple sets of samples run simultaneously (either one barcode, single index, or two, dual index). The samples are then loaded into the sequencer where they are washed over a small, thin surface known as a flow cell. The samples attach to the flow cell via the ligated capture sites. Since there are capture sites on both sides, the molecule can bend over to attach to the flow cell at both ends. A polymerase and dNTPs are introduced to amplify the DNA which is separated again into ssDNA. This process is repeated several times within an area of the flow cell to form a cluster of replicated molecules. The flow cell is then exposed to a polymerase and fluorescent dNTPs. The dNTPs are added one at a time to a cluster which fluoresces a different color depending on the incorporated base until the full sequence has been determined. The Illumina technology allows sequencing from just one side (single-read, SR) or from both ends (paired-end, PE). Paired-end runs, which increase the accuracy of genome mapping, are typically more expensive and more useful for difficult, repetitive genomic sequences or providing information about splice junctions and alternatively spliced transcripts (Williams et al., 2014). PE is not required for DGE analysis but may provide greater coverage during the genomic alignment stage. Each machine offers a maximum achievable number of output sequence reads per lane of a flow cell (with a total of eight lanes per flow cell). Therefore, the number of samples loaded per lane must be divided by the total number of possible reads to determine the number of reads each sample may receive (the HiSeq 4000 typically delivers ∼300 million total reads per lane). A standardized number of reads required per sample to successfully map to the genome, determine gene expression differences, or other experimental parameters has not yet been determined and must be considered on an experiment-to-experiment basis. Though the ENCODE consortium has released guidelines for standardized RNAseq practices they are now out-of-date given the pace the field is moving. However, some general guidelines and standards are available (Williams et al., 2014; Conesa et al., 2016).

#### Data Preparation Pipeline

There is no one standard analysis pipeline for RNAseq projects as they will vary given the data. Here we present a basic overview of some considerations and software for the DGE analysis process and other downstream analyses (Oshlack et al., 2010; Rapaport et al., 2013; Williams et al., 2014; Finotello and Di Camillo, 2015; Conesa et al., 2016). Also, note that CLIP studies may require specialized experimental design and analysis using specific software (Wang et al., 2015; Zhang et al., 2015). For further help and extra information, the SeqAnswers^2^, Biostars^3^, and StackExchange^4^ forums are all excellent resources. A selected list of currently popular tools for RNAseq data processing and analysis are listed in **Table 2**.

**Table 2:** RNAseq preprocessing and analysis tools currently in common use.

Tool type	Tool name	Reference
Quality Control	FastQC	Andrews, 2010
	RNA-SeQC	DeLuca et al., 2012
	RSeQC	Wang et al., 2012
	ShortRead	Morgan et al., 2009
Trimming, Demultiplexing	FASTX-Toolkit	http://hannonlab.cshl.edu/fastx_toolkit
	Stacks	Catchen et al., 2011
	Trimmomatic	Bolger et al., 2014
	TrimGalore	http://www.bioinformatics.babraham.ac.uk/projects/trim_galore/
General aligners, Psuedoaligners, *De novo* Annotators	Bowtie2	Langmead and Salzberg, 2012
	Kallisto	Bray et al., 2016
	Novalign	http://www.novocraft.com
	SOAP2	Li R. et al., 2009
	STAR	Dobin et al., 2013
	Tophat2	Kim D. et al., 2013
Post-Alignment Processing, QC, Counting, and Visualization	htseq-count	Anders et al., 2013
	IGV	Thorvaldsdottir et al., 2013
	RNA-SeQC	DeLuca et al., 2012
	RSeQC	Wang et al., 2012
	Rsubread	Liao et al., 2013
	SAMtools	Li H. et al., 2009
Differential Expression Analysis	edgeR	Robinson et al., 2010
	DESeq2	Anders et al., 2013
	baySeq	Hardcastle and Kelly, 2010
	Cuffdiff2	Trapnell et al., 2013
	DEGseq	Wang et al., 2010
	EBSeq	Leng et al., 2013, 2015
	voom	Law et al., 2014

The first major consideration before beginning RNAseq analysis is that the process requires a considerable amount of computing power. The amount of power needed will vary depending on the size and type of experiment. As a general minimum guideline: Large disk space (1–5 TB), RAM (at least 8–32 + GB, higher is preferred for large datasets), and a multi-core CPU (8 cored preferred, higher is better since it is often the source of bottleneck) are essential for fast computation; the processing can easily take days on a slower machine and will thus be unavailable for any other use during this time. GPU computing has not currently been optimized for RNAseq. Analysis may be performed using R/Bioconductor packages^5^, specialized software with graphical interfaces (e.g., Galaxy) (Afgan et al., 2016), programming and scripting languages (e.g., R, Matlab, Python, Ruby, Perl, Java etc.), and terminal commands on a UNIX-based operating system such as Ubuntu. Analysis and preprocessing is possible on Windows operating systems but may be difficult to set up because a number of bioinformatics tools have been developed for terminal/command line interfaces that are not normally compatible with the Windows environment. A Linux setup is the most convenient for analysis but it is possible to set up some tools on Windows using the Cygwin terminal. Finally, a useful source for installing bioinformatics tools on UNIX systems can be found here: https://www.biostarhandbook.com/tools/how-to-install-everything.html.

RNAseq preprocessing and analysis roughly falls into the following pipeline:

(i) *Process reads obtained from the sequencer*: The sequencer will return the sequencing data in the form of FASTQ files. For QC, files should be analyzed by a QC tool such as FastQC (Andrews, 2010), ShortRead (Morgan et al., 2009), or RNAseq specific tools RSeQC (Wang et al., 2012) and RNA-SeQC (DeLuca et al., 2012). These tools return a series of graphs and metrics that can be used to evaluate whether or not any data cleaning is necessary. Each FASTQ file lists sequences accompanied by a base quality score (Q score) indicating the probability of an incorrect base assignment for that position in the sequence. The average Q value can be detected using a QC tool. A good sequencing read will have a mean Q value over 30. However, many sequences will have a drop in Q value toward the 3′ end of the sequence and may need trimming by a few bases if they are extremely poor quality. Although trimming reads might be necessary in some special cases, there has been recent work to suggest that overtrimming can affect DGE estimates at later analysis stages (Williams et al., 2016). Finally, the adapter sequence might need to be removed if it was detected during sequencing. Trimming and filtering can be accomplished with the command line or a tool like the FASTX-Toolkit^6^ or Stacks (Catchen et al., 2011). For a more thorough review of QC metrics see (Li et al., 2015).
(ii) *Align reads to reference genome*: Aligners generally fall into two basic categories - those that emphasize speed or those that emphasize sensitivity. Fast aligners include STAR (Dobin et al., 2013), Bowtie2 (Langmead and Salzberg, 2012), and SOAP2 (Li R. et al., 2009). More sensitive aligners include Novalign^7^ and SHRiMP2 (David et al., 2011). TopHat2 (Kim D. et al., 2013), is one of the most widely used alignment tools though it takes a significant time to run. A step-by-step guide to Tophat2 usage can be found in (Anders et al., 2013). For a comparative list of aligners see (Fonseca et al., 2012; Engstrom et al., 2013; Baruzzo et al., 2017). The output of aligned reads is usually stored in either SAM or BAM file formats which can be processed with SAMtools (Li H. et al., 2009). The SAM format is a readable formatted file that can be examined visually. The BAM format is a compressed SAM file that can be processed much more quickly but is not human readable.
(iii) *Generate BAM statistics, visualize aligned reads*: At this stage, it is important to determine the percentage of mapped reads (generally greater than 70% for a successful alignment). The percent of rRNA reads present is also an important metric. Theoretically, the rRNA removal step in library preparation should have removed the rRNA but this step is not 100% efficient. All of the above statistics can be generated either using SAMtools, RSeQC, or RNA-SeQC. The next step is visualization of the alignment. Although SAM files are readable, it is much easier to use a genome visualization tool to confirm the success and quality of the alignment. There are many tools available for this purpose such as IGV (Thorvaldsdottir et al., 2013). SAMtools can be used to convert SAM files into position/index-sorted BAM files so they can be visualized. Visualization should be used to confirm the success of the alignment such as correct mapping over the exon-exon junctions. As a final QC check, if certain transcripts are already known to have differential expression, the genome visualizer may be used to confirm that these expression differences are seen in the aligned RNA samples.
(iv) *Obtain raw read counts and normalize*: After the RNA has been aligned to the genome, raw read counts can be generated for each transcript. HTSeq can be used for this purpose using the htseq-count tool (Anders et al., 2015). In the R/Bioconductor environment, the Genomic Features and Genomic Alignment packages (Lawrence et al., 2013) or Rsubread can be used (Liao et al., 2013). Rsubread is able to count multi-mapped reads which may be useful for DGE analysis. Typically, the next stages of analysis will be the comparison of differentially expressed genes. The issue is that raw counts cannot be compared to each other, either within the same library or comparing samples between different libraries, without subsequent normalization. This is because of an inherent bias in the sequencing process that results from either the depth of sequencing or the length of a transcript. To overcome this issue, a number of different normalization strategies have been developed such as normalization by library size or transcript length (Dillies et al., 2013; Zyprych-Walczak et al., 2015; Lin et al., 2016). One of the first strategies employed is RPKM – reads per kilobase transcript per million reads (Mortazavi et al., 2008). FPKM was introduced later for paired-end data and employs the same principle except that it accounts for the fact that two reads will be mapped to a fragment. Although RPKM can be used for within library comparisons, it is not appropriate for comparisons between libraries because of inconsistent average RPKM (Oshlack and Wakefield, 2009). Other normalization methods that allow between-library comparisons include those that divide raw counts by the median, quantiles (Law et al., 2014), total counts, upper quantile, or factor size as in the R package DESeq2 (Love et al., 2014). See review cited above for a comparison of normalization strategies.

### Downstream Data Analysis

In order to model counts between different groups of genes the distribution of the data must be determined. Since counts from RNAseq data are discrete rather than continuous only specific distributions can be used to model the data. The negative binomial model is currently popular because it corrects for errors that result from modeling with the Poisson distribution and is used by both the DESeq2 and EdgeR (Robinson et al., 2010) packages. For a detailed step-by-step guide to using both of these packages see (Anders et al., 2013). Both DESeq2 and EdgeR can be used for DGE analysis of RIP-SEQ data. However, an alternative package called RIPSeeker has been developed recently that uses peak-calling, a strategy employed in ChIP-SEQ/CLIP-SEQ data analysis, and boasts better modeling accuracy than DESeq2 and EdgeR for RIP-SEQ data (Li et al., 2013). RNAseq may also be used to detect isoform level quantification or differential expression for a given RNA (Will et al., 2013). A few software packages exist that may aid in this analysis include EBSeq (Leng et al., 2013, 2015) and MISO (Katz et al., 2010). There are also a number of other software available for DGE analysis that uses different types of modeling (Rapaport et al., 2013; Soneson and Delorenzi, 2013; Seyednasrollah et al., 2015). Soneson and Delorenzi (2013) have provided a detailed analysis of the different methods and their performance depending on the features of the data (sample size, degree of differential expression etc.). It is suggested to try a few different methods to see how different the generated differential expression values are for each gene.

In addition to DGE analysis, there are a number of options available to further analyze the data. Here we briefly list a few approaches. After obtaining the list of proteins and RNA, the data may be subjected to gene ontology (GO) clustering to find broad associations in the data. There are a number of GO clustering software options available but DAVID is among the simplest to use (Huang da et al., 2009a,b). Given a specific list of genes, DAVID will associate the data with gene ontologies in the hierarchy and cluster the most representative terms. The full GO ontology as well as a smaller version is available. DAVID also includes annotation for pathways, protein domains, and protein interactions providing a convenient means to get a broad view of the data. GOseq is another alternative which takes into account the transcript length bias mentioned above (Young et al., 2010).

Network analysis can be extremely helpful in visualizing complex data. Cytoscape and its associated plug-ins give the user a high amount of customization and flexibility to visually map connections between genes associated in different pathways, molecular interaction networks, and disease databases (Shannon et al., 2003). Some plugins also allow input of gene expression data as an extra visual dimension to the data. For example, the Enrichment Map plugin from the Bader Lab allows the user to take GO clustered data from DAVID and visualize it as a network. Enrichment Map helps to simplify data produced by DAVID in a visual format for easier interpretation (Merico et al., 2010, 2011). This network-based approach is especially useful for modeling interaction networks of proteomics data and has been used to characterize molecular networks in synaptic plasticity (Pocklington et al., 2006).

Clustering (K-means, hierarchical clustering etc.) is also used to find patterns in gene expression data. Clustering will group a set of genes into categories based on the similarity of their expression levels. Used in tandem with a heatmap, expression changes can be visualized on a color scale while simultaneously grouped by similarity in expression level changes based on a chosen similarity metric (D’haeseleer, 2005). The Genesis software provides an easy way to perform various clustering methods^8^. Many clustering strategies assume the data is normally distributed which is not usually the case for RNAseq data. One option is to apply a transformation to the data so that the counts are more normally distributed and then apply the method (Zwiener et al., 2014). Alternatively, there are a few methods that have been recently developed that apply clustering strategies to either Poisson or negative binomial distributions which much more closely approximate RNAseq data (Witten, 2011; Si et al., 2014). Principal components analysis (PCA), support vector machines (SVM) and other tools for dimensionality reduction and classification analysis can also be applied to RNAseq data. For review, see Tan et al. (2014).

## Combined Transcriptomics and Proteomics

The overall goal of RNA sequence identification and bioinformatics analysis on synaptic fractions is to determine how the synapse is remodeled under dynamic physiological and disease conditions. Since expression of proteins with coordinated functions can rapidly change the efficacy of a synapse, proteomics analysis is thus a critical counterpart to RNA sequencing experiments (Martin and Zukin, 2006; Zukin et al., 2009; Fernandez-Moya et al., 2014; Rosenberg et al., 2014). The increased sensitivity and accuracy of high-throughput methods have now made it feasible to conduct RNASeq studies and follow up by mass spectrometry. While the application of Omics to synaptic data is slowly growing (Collins et al., 2006; Fernandez et al., 2009; Geschwind and Konopka, 2009; Fritzsche et al., 2013; Ch’ng et al., 2015; Hussain and Bashir, 2015; Broek et al., 2016; Kenney et al., 2016; Loos et al., 2016; Niere et al., 2016) few studies combining multiple Omics have been performed in a synaptic context (Valor and Grant, 2007; Kitchen et al., 2014). To date, combined approaches have been used to determine differences in neuronal cell types (Sharma et al., 2015), the effects of oxidative stress in synaptosomes (Flynn et al., 2012), the age-specific differences in brains of old and young rats (Ori et al., 2015), differences in stages of embryonic development (Hartl et al., 2008), cell type-specific proteins enriched in the brain over other tissues (Sjostedt et al., 2015), and the molecular dynamics of Rett Syndrome (Li et al., 2016). One of the most important findings from the combined omics approach is the confirmation that protein and RNA levels from the same tissue or single-cell do not always correlate (Maier et al., 2009; Olivares-Hernandez et al., 2011; Haider and Pal, 2013). Therefore, such an integrated approach will allow more accurate modeling of the dynamic molecular interplay that underlies synaptic function such as post-transcriptional regulation by non-coding RNA as well as proteomic regulation through post-translational modifications.

### *De novo* Protein Synthesis Assays

The separation of established protein from de novo protein synthesis has been challenging. A variety of methods for isolating protein populations may be employed and are reviewed in **Table 3**. Methods such as BONCAT (bioorthogonal non-canonical amino acid tagging) were recently developed as a means of selectively isolating newly synthesized proteins from a larger population (Dieterich et al., 2006, 2007). Utilizing click chemistry, newly synthesized proteins incorporate a non-canonical amino acid using the cell’s own translational machinery which can be conjugated to biotin. One of the great advantages of this method is that endogenous proteins are labeled as opposed to introducing exogenous reporter proteins. These proteins can then be isolated from the rest of the population using affinity chromatography and analyzed by mass spectrometry. Instead of non-canonical amino acids, SILAC (stable isotope labeling by amino acids in cell culture) uses heavy or light isotopes of arginine and lysine (Ong et al., 2002). One population of cell culture will be grown with media containing the heavy isotope and a second population will use the light isotope. The proteins from both populations are then combined and analyzed by mass spectrometry. A protein of interest can then be compared between the two different cell cultures – one will be heavier due to incorporation of the heavy isotope and its abundance levels can be compared to the same protein in the other cell culture thus demonstrating a change in expression between two cellular conditions. Though both BONCAT and SILAC address different kinds of questions, there have been a number of recent developments of SILAC such as pulsed SILAC (pSILAC) that allows for the determination of newly synthesized proteins (Chen et al., 2015). There have also been a few recent attempts to combine BONCAT and SILAC together (Genheden et al., 2015; Kenney et al., 2016). Kenney et al. (2016) applied this technique to cell culture by first introducing medium or heavy arginine to the cells (SILAC) followed by the addition of a non-canonical amino acid to isolate newly synthesized proteins (BONCAT). This combined approach allowed for the comparison of specific newly synthesized proteins rather than the entire population. Furthermore, another approach called BONLAC combines the two methods and optimizes BONCAT conditions so that it may be performed in intact brain slices (Bowling et al., 2016). In addition to labeling with a non-canonical amino acid (AHA, a methionine analog) as in BONCAT, Bowling et al. (2016) also labeled with medium or heavy arginine thus allowing them to selectively isolate *de novo* synthesized proteins within a short time window. An *in vivo* BONCAT approach was also developed by Liu and Cline (2016) which allowed them to assess the effect of an FMRP knockdown on protein synthesis-dependent behavioral plasticity in the *Xenopus* visual system. The evolution of this method has greatly expanded our knowledge as it now allows the detection of activity-dependent *de novo* protein synthesis *in vivo*.

**Table 3:** Summary of methods for identifying protein–protein interactions.

Assay type	Technique	Advantages	Disadvantages	Reference
Direct RNA-protein interactions	Immunoprecipitation	Single molecule (IP) or complex (Co-IP)	Antibody-based, non-specific binding, difficult to detect proteins with low expression, cannot identify transient interactions	Markham et al., 2007; Sutherland et al., 2008; Free et al., 2009; Mikula et al., 2015
	Pull-down	Tag-based, does not require antibody	Tag may be difficult to engineer and may alter protein function, difficult to detect proteins with low expression, cannot identify transient interactions	
Labeling methods	Label transfer protein interaction	Isolation of transient protein–protein interactions, interaction within physiological context	Difficult to balance dissociation timing with label transfer molecule	Liu et al., 2007
	BioID	Overcomes difficulties in Co-IP and pull-downs	Best suited for culture work, fusion protein may interfere with protein interactions, biotin may alter properties of protein/interacting partners	Roux et al., 2012
Crosslinking methods	Analysis of oligo(dT)-purified mRNPs	Can observe dynamic changes in RNA–protein interactions	Cannot identify non-Poly(A) proteins or microRNAs	Baltz et al., 2012
*In vitro* binding	Modified phage display	Compliment to other protein-protein interactions methods	Technically challenging, will pick up non-physiological interactions	Di Niro et al., 2010
	RNA bait quantitative proteomics	Compliment to other protein–protein interactions methods	Will pick up non-physiological interactions	Butter et al., 2009
	Size-exclusion quantitative proteomics	Identify transient interactions, does not require any tags	Will pick up non-physiological interactions	Kirkwood et al., 2013

## From Statistical Models to Scientific Models: Experimental Validation and Visualization of Identified Proteins and RNA

**FIGURE 4:**
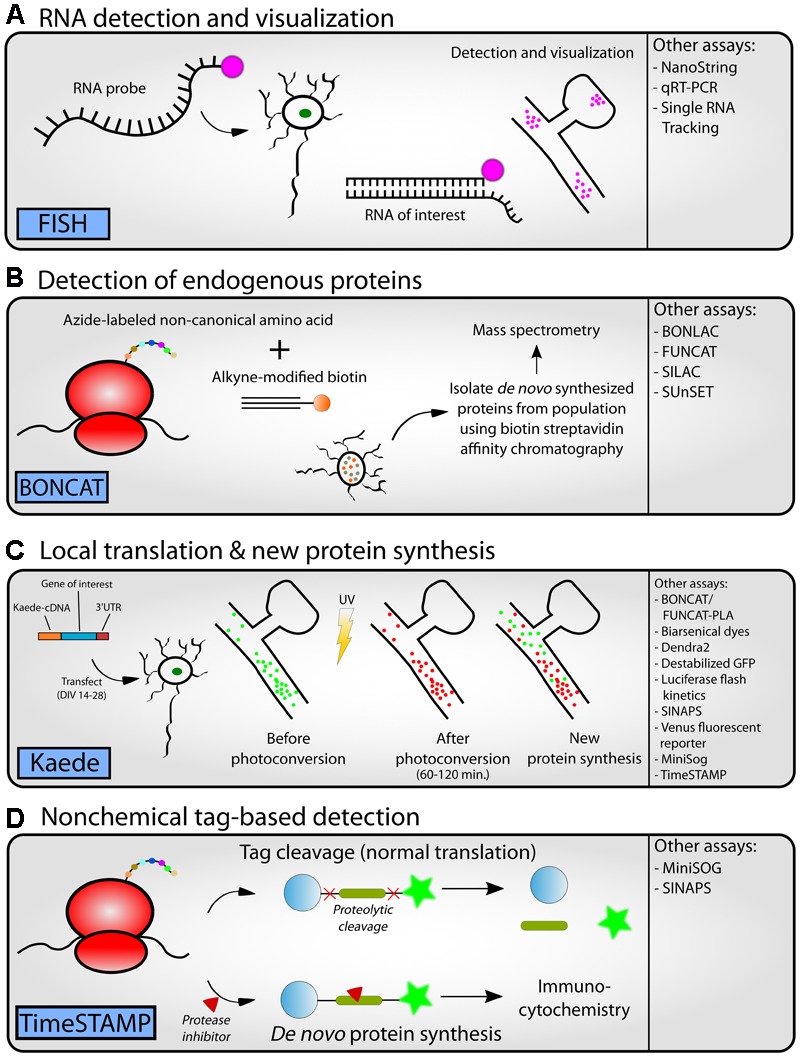
**Workflow for experimental follow-up.** Following the completion of high-throughput methodologies, researchers can perform a variety of different types of experiments to follow-up predictions seen in the data. Representative assays are depicted in cartoon form (**A–D**, left panel) and alternative approaches (**A–D**, right panel) or references can be found in **Table 4** or the text. **(A)** RNA detection and visualization using fluorescence *in situ* hybridization (FISH) in neurons. An RNA probe complementary to an RNA of interest is designed. A fluorescent tag is added to the probe for detection downstream. The probe is introduced to a neuron population. Once the RNA enters the neuron, it binds to the RNA of interest. Cells can then be fixed and visualized by fluorescence microscopy to visualize the location of the RNA within a tissue or cell culture system. **(B)** Detection of endogenous proteins using bioorthogonal non-canonical amino acid tagging (BONCAT). An azide-labeled non-canonical amino acid is introduced to a cell population. During translation, this amino acid is conjugated to alkyne-modified biotin using click chemistry, thus tagging a newly synthesized protein. Affinity chromatography is used to isolate these biotin-labeled *de novo* synthesized proteins from the greater protein population. The isolated proteins can be identified using mass spectrometry. **(C)** The photoconvertible fluorescent protein Kaede can be used to visualize local translation/new protein synthesis of a protein of interest. A vector containing Kaede-cDNA is introduced to a cell culture population. Prior to photoconversion, Kaede fluoresces green revealing the current population of a protein of interest. After application of UV light, Kaede fluoresces red. Any further green signal that appears later is indicative of new translation of the protein of interest. **(D)** Options for non-chemical tag-based detection include time-specific tagging for the age measurement of proteins (TimeSTAMP). A fusion construct is generated between the protein of interest and an epitope tag flanked by a cassette for the hepatitis C virus protease. After translation, the protease cleaves itself and the epitope tag away from the protein of interest. The protease inhibitor BILN-2061 may be added to the cells at any time to inhibit the proteolytic cleavage. Thus, the protein of interest can retain its tag. Using this system, researchers can separate new and old protein synthesis after a specific time point.

One of the greatest advantages of performing unbiased screens such as RNAseq and mass spectrometry is the identification of novel interactions between mRNAs and/or proteins. The wealth of data generated from such high-throughput experiments can be used to build general models to guide the direction of future scientific research. For example, constructing protein–protein interaction (PPI) networks from proteomics data can be used to guide experimental research leading to new target identification in animal models of disease. (for further explanation of PPI network construction and analysis see Raman, 2010). For example, this technique was recently used by Niere et al. (2016) with the reasoning that many individuals who have neurological disorders with dysregulated protein synthesis due to overactive mTOR signaling also suffer from epilepsy (Crino, 2008; Pun et al., 2012; Brewster et al., 2013; Wong, 2014; Sosanya et al., 2015). To identify common proteins associated with epilepsy, Alzheimer’s disease, and Autism Spectrum Disorders, all disorders with overactive mTOR signaling, a PPI network was established that identified 5 “hub proteins” based on its high level of connectivity with other proteins in the network. One hub protein was Parkinson Protein 7 (Park7 or DJ-1), a protein that has many functions but most recently has been identified as an RBP(van der Brug et al., 2008). Importantly, the investigators went on to show that Park7 protein synthesis was regulated by mTOR and that it is overexpressed at synapses in a mouse model of Tuberous Sclerosis Complex, a form of ASD with overactive mTOR. Below we review techniques that may be used to visualize the localization and relative quantity of protein and/or RNA to validate models generated from high-throughput methodologies (**Table 4**; **Figures 4A–D**). While most of these techniques suffer from the single protein/RNA approach, they do provide a powerful means of validating findings that may lead to new target identification of diseases with dysregulated protein synthesis.

**Table 4:** Visualization and detection techniques for RNA and protein downstream of high-throughput experiments.

Detection of a reporter or endogenous RNA/protein	Technique	Detection chemistry	Detection Type	Selected References
Endogenous (RNA)	(F)ISH	Hybridization probe; fluorescent antibody	RNA localization	Lecuyer et al., 2007; Swanger et al., 2011; Cajigas et al., 2012
	Single RNA tracking	Fluorescent dyes	RNA localization and translation	Katz et al., 2016
	qRT-PCR	Fluorescent DNA intercolator	Relative RNA quantitation	Raymaekers et al., 2009
	NanoString	Hybridization probe	Relative RNA quantitation	Kulkarni, 2011; Cajigas et al., 2012
Endogenous (Protein)	BONCAT	Biotin	*De novo* synthesis	Dieterich et al., 2006, 2007
	SILAC	Heavy/light chain amino acid isotopes	*De novo* synthesis	Ong et al., 2002
	BONLAC	Biotin + heavy/light chain amino acid isotopes	*De novo* synthesis	Bowling et al., 2016
	FUNCAT	Biotin, fluorescent antibodies	De novo synthesis	Dieterich et al., 2010; Tom Dieck et al., 2012; Kos et al., 2016; Liu and Cline, 2016
	BONCAT/FUNCAT-PLA	Biotin, fluorescent antibodies	*De novo* synthesis	tom Dieck et al., 2015; Workman et al., 2015; Niere et al., 2016
	SUnSET	Fluorescent antibody	*De novo* synthesis	Schmidt et al., 2009; Batista et al., 2016
Reporter construct (Protein)	Destabilized GFP (dGFP)	Fluorescent protein (fusion construct)	*De novo* localized synthesis	Li et al., 1998; Aakalu et al., 2001
	Kaede	Photoactivatible GFP-like fluorescent protein (PAFPs)	*De novo* localized synthesis	Ando et al., 2002; Lukyanov et al., 2005; Raab-Graham et al., 2006; Workman et al., 2015
	Dendra2	Photoactivatible GFP-like fluorescent protein (PAFPs)	*De novo* localized synthesis	Gurskaya et al., 2006; Chudakov et al., 2007; Wang et al., 2009; Lee et al., 2011
	Venus fluorescent reporter	Fluorescent protein (fusion construct)	*De novo* localized synthesis	Tatavarty et al., 2012; Ifrim et al., 2015
	Biarsenical probes (FlAsH, ReAsH)	Fluorescent dyes	*De novo* localized synthesis	Ju et al., 2004;Martin et al., 2005; Rodriguez et al., 2006
	TimeSTAMP	Epitope tag	*De novo* localized synthesis	Lin et al., 2008; Lin and Tsien, 2010
	MiniSOG	Fluorescent protein (fusion construct)	*De novo* localized synthesis	Shu et al., 2011
	Luciferase Flash Kinetics	Luciferase (fusion construct)	*De novo* localized synthesis	Na et al., 2016
	SINAPS	Fluorescent protein (fusion construct)	Real-time translation dynamics	Wu et al., 2016

### Visualization and Quantification of RNA Localization

#### *In situ* Hybridization

The first requirement for a protein to be synthesized in dendrites is that the mRNA coding for that protein is either targeted to the dendrites in response to activity or constitutively resides in dendrites. Some of the earliest work in this field utilized various methods such as *in situ* hybridization (ISH) and microarrays in conjunction with subcellular fractionation to catalog the RNAs present at the dendrite. One of the greatest advantages ISH is that it does not rely on subcellular fractionation – the transcript can be identified in fully intact tissue or cell culture. Thus, ISH and its related methods are very useful for confirming results obtained from subcellular fraction preparations. ISH has been used to characterize the localization and distribution of the RNA transcripts for the calcium/calmodulin-dependent protein kinase II alpha (CaMKII), microtubule-associated protein 2 (MAP2) and fragile X mental retardation protein (FMRP), some of the earliest localized transcripts discovered in the field (Hinds et al., 1993; Paradies and Steward, 1997; Miyashiro et al., 2003). The development of fluorescence *in situ* hybridization (FISH) has allowed researchers to visualize distribution patterns and quantify the amount of localization at a much higher resolution. There have been significant technical improvements made to FISH over the years which provide greater resolution and better quantification (Swanger et al., 2011). The best example of how technical improvements in FISH have changed dogma was first described by Krause and colleagues using mRNA localization in the *Drosophilia* embryo as a model. Prior to this study the estimated number of mRNAs that had distinct subcellular localizations in the embryo was ∼1-10%. Lecuyer et al. (2007) screened roughly 25% of the genome and found ∼ 71% of mRNAs screened had unique subcellular distribution patterns. The authors went on to suggest that since most of the transcribed mRNAs have a distinct subcellular localization these data imply that most cellular processes are mediated through mRNA localization (Lecuyer et al., 2007). Importantly, a similar finding was suggested by Schuman and colleagues in neuronal dendrites. They also employed FISH to verify high-throughput RNA sequencing data (Cajigas et al., 2012). Herein they identified 8,379 transcripts. By subtracting transcripts related to glial cells, interneurons, nuclei, blood vessels, and mitochondria, they suggest that 2,550 transcripts are localized in axons or dendrites. High-sensitivity FISH was then used to validate the localization of 50 of these transcripts. This work identified many known synaptic transcripts as well as newly discovered transcripts that had not been previously detected. In light of these findings synapse remodeling of synapses during synaptic plasticity is due to dendritic over somatic mRNA translation (Cajigas et al., 2012). Finally, a more recent developed in FISH allows for the visualization of single RNA molecules at high resolution. Singer and colleagues have developed a method that allows the tracking of single RNA molecules during translation. The researchers labeled ribosomes and mRNA molecules and correlated their signals to determine if the mRNA was undergoing active translation (Katz et al., 2016). Thus, collectively RNA sequencing data combined with high resolution FISH, for the first time, is allowing investigators to catalog mRNAs localized to site specific dendritic compartments leading to new testable hypothesis regarding memory allocation for information storage.

#### Quantification of mRNA

While FISH by itself provides subcellular localization of specific mRNAs, quantification of mRNAs in the soma versus dendritic compartments has been challenging. The development of the NanoString nCounter Gene Expression Assay has helped resolve that issue (Kulkarni, 2011). NanoString enables the detection and quantification of up to 800 mRNA molecules (without any conversion to cDNA or PCR amplification) using colored probe pairs. Cajigas et al. (2012) used NanoString technology to answer the long-standing question of whether select mRNAs segregate, with some enriched in the soma and some enriched in dendrites and axons. While this finding had been suggested by quantitative RT-PCR comparing hippocampal synaptosomal mRNA to total lysate mRNA (Raab-Graham et al., 2006) the concern of somatic contamination in the synaptosomal fraction tempered the interpretation of this finding. Still, in cases when mRNA levels are low-abundance or quick validation is a necessity, qRT-PCR remains a valid method for confirming RNAseq or microarray data, or quantifying relative abundance levels of a specific transcript between samples (Raymaekers et al., 2009).

### Visualization of Protein Localization and New Translation

#### SUnSET, FUNCAT, and FUNCAT/BONCAT-PLA

Labeling newly synthesized proteins has been used to validate high-throughput studies investigating local translation (Buxbaum et al., 2015a,b). Since many of these techniques rely on fluorescent microscopy, it is possible to visualize and distinguish new and old proteins within a particular neuronal compartment. Surface sensing of translation (SUnSET) utilizes puromycin, a ribosome elongation inhibitor and aminoacyl-tRNA synthetase analog, to monitor the translation of *de novo* protein synthesis (Schmidt et al., 2009). SUnSET uses monoclonal antibodies to detect the incorporation of puromycin into the polypeptide chain during translation. Subsequent detection and visualization by either flow cytometry or fluorescent microscopy thus indicates new protein synthesis.

Single-molecule imaging of nascent peptides (SINAPS) is another alternative approach for monitoring localized transcripts *in vivo* which allows one to observe the real-time translation of nascent mRNA molecules at different translational stages (Wu et al., 2016). In order to strengthen the relatively weak signal of the nascent peptide and to distinguish it from background, SINAPS draws upon SunTag, another recent method that allows for the amplification of fluorescent intensity by recruiting multiple copies of GFP to a target protein (Tanenbaum et al., 2014).

The FUNCAT (fluorescent non-canonical amino acid tagging) assay is similar to the BONCAT technique described earlier which labels newly synthesized proteins using non-canonical amino acids and click chemistry to conjugate biotin. FUNCAT, however, uses fluorescently tagged amino acids to allow for the identification of newly synthesized proteins *in situ* by fluorescence microscopy (Dieterich et al., 2010; Tom Dieck et al., 2012; Kos et al., 2016). The drawback to FUNCAT is the fact that while it can identify whole populations of *de novo* synthesized proteins, it cannot identify specific ones. To overcome this difficulty, a modification was developed using a proximity ligation assay (PLA). The use of FUNCAT in tandem with PLA allows for the identification of a newly synthesized protein of interest (tom Dieck et al., 2015). FUNCAT-PLA – also called BONCAT-PLA in (Niere et al., 2016) – utilizes a biotin antibody to identify a protein of interest that has incorporated a non-canonical amino acid, signifying new protein synthesis. Another antibody is used to identify the protein of interest itself. When the secondary antibodies (pre-conjugated to a specific oligonucleotide sequence) for the two primary antibodies are in close enough proximity, they are ligated together and undergo rolling circle replication in the presence of fluorescent nucleotides to produce a signal. Alternatively, the Puro-PLA assay utilizes puromycin, a molecule that disrupts translation resulting in the release of the newly synthesized protein which can then be identified with an anti-puromycin antibody (Buhr et al., 2015; tom Dieck et al., 2015).

Recently, FUNCAT/BONCAT-PLA has been used to assess the role of mTOR complex 1 (mTORC1) in synapse modification *in vivo.* mTORC1 is a protein complex that regulates local dendritic translation (Tang et al., 2002; Cammalleri et al., 2003; Stoica et al., 2011). In a recent study, Niere et al. (2016) performed mass spectrometry on different subcellular fractions of neurons derived from rat cortices after an intraperitoneal injection with rapamycin, an inhibitor of mTORC1. Notably, the mass spectrometry reports changes in protein expression and does not differentiate between protein synthesis and protein stability. To differentiate between these two cellular mechanisms, they used FUNCAT/BONCAT-PLA to confirm new protein synthesis of select candidates identified by mass spectrometry (Niere et al., 2016). Indeed, FUNCAT/BONCAT-PLA demonstrated that new protein synthesis levels of Snap25 and Gap43 altered in response to treatment with the mTOR inhibitor rapamycin, and were consistent with the observed site-specific changes in protein expression indicated by mass spectrometry. Thus, new protein assays such as FUNCAT/BONCAT-PLA can detect new protein synthesis, providing mechanistic detail that mass spectrometry hints at but does not confirm.

#### Destabilized GFP (dGFP)

While FUNCAT/BONCAT-PLA is useful to detect new protein synthesis it does not provide direct evidence for new protein synthesis in dendrites. For this reason, fluorescent translation reporters fused to dendritic targeting sequences of the proteins of interest still remains the best way to visualize new protein synthesis in dendrites. This approach was first developed by Schuman and colleagues (Aakalu et al., 2001). Destabilized-GFP (dGFP) was developed to address experiments that required a fluorescent reporter for proteins with transient expression (Li et al., 1998). Schuman and colleagues capitalized on the rapid turnover properties of dGFP and created a reporter consisting of cDNA coding for a myristoylated dGFP fused between the 5′ and 3′UTRs of CaMKIIα mRNA. They reasoned that the inclusion of the UTR sequences in their reporter construct would ensure that the mRNA targets the dendrite, the addition of a myristoylation sequence (myr) tethers the reporter to the membrane and thus prevents diffusion, and after photobleaching the neuron, new GFP signal detected in the dendrite is due to mRNA translation. To ensure this was the case, they continuously photobleached the soma so that any new protein synthesized in the soma would not be detected in the dendrites. One caveat that may hinder this assay is if the protein dGFP is fused to have a higher stability than dGFP, thus preventing the rapid turnover of GFP. In spite of this limitation, myrdGFP fused to the appropriate targeting sequences can be used to investigate compartment-specific translation (Aakalu et al., 2001).

#### Kaede, Dendra2, Venus, and Biarsenical Probes

An alternative approach is the usage of photoactivatable GFP-like fluorescent proteins (PAFPs) (Lukyanov et al., 2005) which overcomes the limitation of variability in mRNA stability. Kaede is a protein that allows the separation of new and old translation on the basis of UV-induced photoconversion (Ando et al., 2002). Kaede can be fused to specific proteins with the appropriate dendritic targeting sequences and used to report new translation by comparing mean puncta intensity before and after photoconversion (Leung et al., 2006; Raab-Graham et al., 2006; Leung and Holt, 2008; Banerjee et al., 2009; Workman et al., 2015). Kaede’s tetrameric structure has a distinct advantage when it comes to the tagging of ion channels which are typically tetramers (Raab-Graham et al., 2006). For example, Kaede has been used to monitor the local translation of Kv1.1, an ion channel whose translation is regulated by a microRNA, demonstrating its use and application as a detector of new protein synthesis (Sosanya et al., 2013). The Dendra2 reporter is another photoconvertible fusion protein that functions similarly to Kaede (Gurskaya et al., 2006; Chudakov et al., 2007; Wang et al., 2009; Lee et al., 2011). Dendra2 has a monomeric structure which increases functional capabilities since it can be used in protein fusion constructs. For example, Wang and colleagues fused the Dendra2 coding sequence to the 5′ and 3′UTRs of sensorin mRNA to study local translation at the sensory neuron-motor neuron junction of an *Aplysia* cell culture system (Wang et al., 2009). Sensorin is a peptide neurotransmitter whose UTR sequences drive the mRNA to be concentrated at synapses, thus allowing the researchers to visualize local translation specifically at the synapse. One advantage of using Dendra2 over Kaede and other similar PAFPs is that it is activated in the 488-nm laser range, well clear of the UV-activation range and thus allowing less chance of cellular toxicity. The Venus fluorescent reporter has recently been adapted to visualize the activity-dependent dendritic localization of PSD-95 (Tatavarty et al., 2012; Ifrim et al., 2015). Ifrim et al. (2015) developed a unique fusion construct in which the Venus reporter was inserted after the 5’UTR of the PSD95 mRNA sequence. Used in this way, the Venus reporter (1) fluoresces just prior to and during translation of the PSD95 open reading frame in real-time and (2) visualizes *de novo* protein synthesis of PSD95 specifically in the dendritic spine (Ifrim et al., 2015). Finally, another approach to monitoring local translation is the use of biarsenical fluorescent dyes such as FlAsH and ReAsH (Ju et al., 2004; Martin et al., 2005; Rodriguez et al., 2006).

#### TimeSTAMP, Luciferase Flash Kinetics, and MiniSOG

TimeSTAMP is another method that can be used to monitor new protein synthesis that does not rely on either photoconversion or chemical tags (Lin et al., 2008; Lin and Tsien, 2010). An epitope tag is attached to a protein of interest which can be removed in the presence of a specific protease. Introduction of a drug prevents this process from occurring thus allowing newly synthesized proteins to retain their tags. TimeSTAMP allows the surveying of the whole brain in living animals.

Recently, a luciferase-based approach that utilizes flash kinetics has also been developed (Na et al., 2016). This approach uses a small luciferase protein derived from *Gaussia princeps* whose signal is dependent on the presence of coelenteraizine. Luciferase is fused to the protein of interest so that, upon translation, luciferase will react with coelenteraizine to produce a light signal. The signal decays rapidly (<9 s for the tested Arc-luciferase construct) so translating RNAs can be unambiguously detected even if coelenteraizine is still present. Thus, flashes of light signal indicate *de novo* protein synthesis of the protein of interest. Importantly, this technique utilizes wide-field microscopy, whereas other reporter systems usually require confocal or 2-photon imaging to detect signals above photobleaching (Na et al., 2016), making this assay accessible to a greater number of labs. Moreover, the authors who developed this assay suggest that estimation of the number of proteins synthesized in a local translation hot spot is within reach if combined with SUnSET; however, this is yet to be verified.

There are a growing number of electron microscopy (EM) studies detecting polyribosomes near spine bases that relocate in the spine head upon memory consolidation (Ostroff et al., 2010, 2014, 2017). However, new protein detection at the EM level has been difficult due to a lack of electron dense tags useful for specific protein visualization. Shu et al. (2011) has developed a small fluorescent protein, MiniSOG (mini singlet oxygen generator), that can be fused to a protein of interest and expressed in cells, tissue, and living organisms and detected both at the light level and by EM. Fluorescence photooxidation of DAB can be achieved using fluorescence and ^1^O_2_ from MiniSOG. It’s been suggested that spatiotemporal control of local photogeneration of ^1^O_2_ will rapidly inactivate proteins of interest (Shu et al., 2011), perhaps allowing one to detect site specific translation at the light and subsequently at the EM level. Although, techniques like miniSOG hold promise, detection of specific translation at the EM level is still lacking.

#### Ensuring that Synthesis of the Protein of Interest Occurs in Dendrites and Not the Soma

An additional requirement for local protein synthesis assays is to demonstrate that the synthesis occurs in the dendrites and not in the soma. In a perfect world, researchers would sever dendrites and test for the appearance of newly synthesized proteins within isolated dendritic compartments. While Martin et al. (1997) have successfully demonstrated local synthesis in processes in the absence of a soma in cultured *Aplysia* neurons, it has proven far more difficult in mammalian neurons. Several labs have introduced microlesions to separate the dendrite and the soma in acute hippocampal slices (Kang and Schuman, 1996; Ouyang et al., 1999; Waung et al., 2008). Kang and Schuman (1996) used this approach to show the local synthesis of BDNF occurred in the dendrites with BDNF-mediated potentiation. While this approach appears optimal, it does have its caveats. We have noted that severing dendrites in this way can induce local protein synthesis of some proteins, often confounding the results when assessing activity-dependent local protein synthesis (unpublished observation). Thus, methods have evolved that have adapted time-lapse imaging of fluorescent proteins fused to specific dendritic targeting sequences to measure *de novo* protein synthesis. Such measures as continuously photobleaching the soma have been used to avoid signal from the soma confounding dendritically translated fluorescent proteins (Aakalu et al., 2001). While a significant improvement over previous methods, continuous photobleaching can be toxic to the cell.

The use of photoactivated fluorescent reporters, such as Kaede, does not require constant photoconversion, in most cases, to specifically detect protein synthesis in dendrites. However, these experiments require careful controls to be able to draw this conclusion. As observed by many laboratories, locally synthesized protein synthesis occurs in hot spots of the dendrites (Aakalu et al., 2001; Job and Eberwine, 2001; Kim T.K. et al., 2013; Sosanya et al., 2015). Therefore, measuring the rate of diffusion of the protein of interest by photoconverting part of the dendrite and measuring diffusion over time (i.e., does the photoconverted protein diffuse into the unconverted dendrite) allows one to measure diffusion rates. For example, Raab-Graham et al. (2006) used such an approach by fusing Kaede to the mRNA of Kv1.1 plus its 3′UTR. It was found that Kaede-Kv1.1 did not diffuse, and stayed localized to hot spots within the dendrite. Thus, proteins presumably function close to the site of synthesis and are easily detected in dendrites when fused to photoactivatable proteins.

Additional approaches include only activating specific synapses and measuring the increase in protein expression within the activated part of the dendrite. For example, Huber and colleagues measured Arc expression after local perfusion of the mGluR agonist DHPG in distal dendrites (Waung et al., 2008). This technique relies on the fact that Arc expression occurred only in the region of mGluR activation and that protein made in the soma cannot traffic to the distal dendrites within the limited time that new Arc protein expression was detected.

Finally, one of the more thorough experimental approaches that allows one to test for function of dendritically localized mRNAs is the generation of a mouse where the dendritic targeting sequence of the protein of interest is knocked out. Mayford and colleagues created a specific knockout deleting only the dendritic targeting sequence in the 3′UTR of CaMKIIα. Importantly, ISH demonstrated that CaMKIIα mRNA was absent from the dendritic fields while still present in the soma of the CA1 hippocampal neurons in the knockout mouse relative to the wildtype litter mate. Moreover, knockout mice showed deficits in late long-term potentiation (L-LTP), a cellular model for learning and memory and at the behavioral level during a memory consolidation test (Miller et al., 2002). While these data are impressive and striking, recent data suggesting that RNA-binding proteins that compete for mRNAs may confound the interpretation of these results (Sosanya et al., 2013). The removal of a dendritic targeting sequence within the 3′UTR containing RBP motifs could possibly free up RBPs to bind to lower affinity target mRNAs which may affect synaptic plasticity and/or behavior. Still, with the development of tools like CRISPR, where making knockouts are quicker and more cost effective, site-specific knockout of proteins will facilitate the physiological relevance of localized translation of specific targets.

## How High Throughput Assays May Answer Long-Standing Questions in the Field of Learning and Memory By Identifying Novel Proteins Involved in Synaptic Plasticity

**FIGURE 5:**
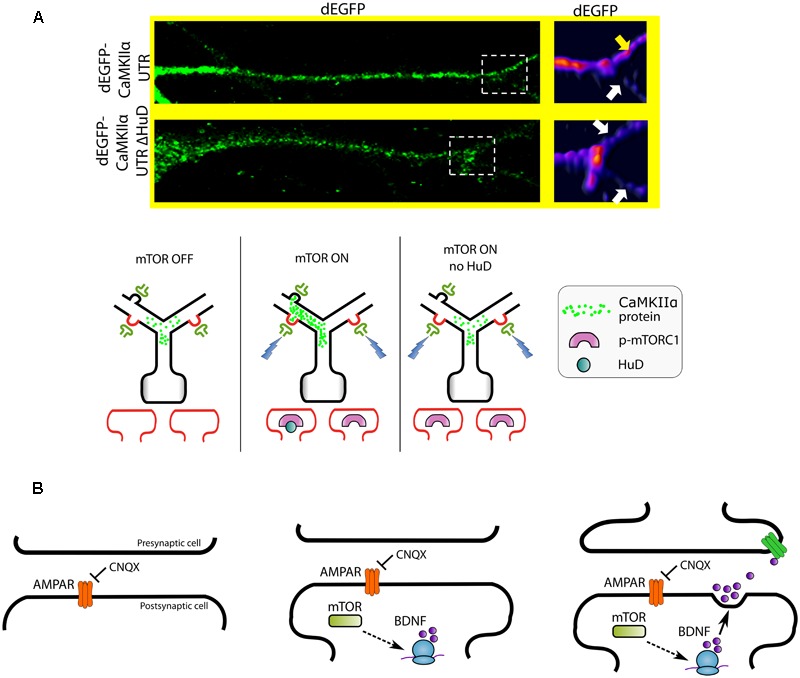
**Approaches to functional validation of high-throughput experiments. (A)** Example of characterization of locally translated mRNAs involved in synaptic tagging and capture hypothesis. RNA sequencing of RNA-binding protein targets may show site specific expression based on the localized trafficking of the RNA-binding protein. (Top) For example, HuD target CaMKIIα mRNA selectively targets one dendritic branch over the other requiring HuD binding, as identified by the translational reporter construct where the coding sequence of dEGFP is fused to the 3′UTR of CaMKIIα mRNA. Deletion of the HuD binding site in the 3′UTR prevents CaMKIIα from entering into the branches and accumulates at the branch point as indicated in the branches pseudo-colored as a measure of intensity. Figure from Sosanya et al., 2015. (Bottom) From these data the working model of HuD’s role in the synaptic tagging and capture hypothesis is illustrated. Panels represent different states of a neuron and dendritic branch point. Protrusions from top branches indicate dendritic spines. Green shapes adjacent to the spines represent presynaptic neurons. Dendritic spines of interest (red) are shown in further detail below each image of the neuron within a panel. (*mTOR OFF)* With no signal, CaMKIIα translocation does not exhibit branch preference. (*mTOR ON*) When mTOR is active, HuD targets its mRNAs into the tagged synapse. Presynaptic signals (blue lightning bolts) trigger translocation of CaMKIIα mRNA to an active synapse. (*mTOR ON or no HuD*) In the absence of HuD or mTOR, CaMKIIα does not show branch preference. **(B)** Model of transsynaptic signaling based on data from (Henry et al., 2012). CNQX-mediated inhibition of AMPA receptors resulting in mTOR activation. mTOR-activation-dependent BDNF synthesis then results in BDNF secretion and binding to presynaptic terminals.

The usage of high-throughput experiments has the potential to point researchers into new directions to answer many long-standing questions in the field of learning and memory. As mentioned above, Niere et al., recently showed that 75% of the PSD changes in protein expression within an hour of mTOR inhibition *in vivo* (Niere et al., 2016). Although this data represents the overall population of dynamic protein changes, individual proteins from this data can be assayed to determine site-specific localization of their mRNAs or local synthesis. Moreover, predictions can be made to identify putative translation regulatory factors such as microRNAs and RBPs based on their mRNA sequences. **Figure 5** outlines a few recent publications that address important questions in neuroscience. Here we provide examples of how others address these questions to assist in RNAseq and proteomic follow up studies.

### Synaptic Tagging and Capture

The synaptic tagging and capture hypothesis (Frey and Morris, 1997; Redondo and Morris, 2011) proposes that synapses undergoing long-term changes to synaptic efficacy are somehow “tagged” in a way that allows localized proteins in one synapse to be “captured” from a protein pool to activate nearby synapses. Sosanya et al. (2015) has shown that mTOR may serve as one such tag. They demonstrated that dendritic branch-specific expression of the CaMKIIα mRNA is mediated by the RNA binding protein HuD. mTOR stabilizes the mRNA and prevents degradation. Thus, mTOR serves as the tag while HuD captures CaMKIIα mRNA to promote its branch specific expression (**Figure 5A**). Data provided by high-throughput experiments that map RNA binding sites such as CLIP-SEQ used in concert with protein visualization techniques may reveal other HuD target mRNAs that have branch specific expression.

### *Trans*-synaptic Signaling

An important but often overlooked feature involved in neuronal homeostasis is *trans*-synaptic signaling, the process by which proteins secreted from the postsynaptic spine can trigger retrograde activation of presynaptic neurons. Recently, it was shown that mTORC1 activation leads to the expression of BDNF which acts as a retrograde messenger to stimulate further neurotransmitter release from the presynaptic terminal (Henry et al., 2012) (**Figure 5B**). A more thorough construction of pre- and postsynaptic signaling/regulatory networks through high-throughput experiments will be invaluable in determining the nature of this postsynaptic engagement.

## Conclusion

In the last decade, we have seen a vast expansion in the application of high-throughput experiments in providing models for the physiological relevance of locally synthesized proteins. This expansion includes the earliest microarray studies on synaptic fractions, where it was considered fantasy to suggest ∼400 mRNAs are localized in dendrites, to large scale RNA sequencing data suggesting closer to 2500 mRNAs reside in dendrites. Collectively, these data are expanding our knowledge of how cellular processes of learning and memory occur. Protein synthesis-dependent synaptic plasticity has been described by many investigators utilizing cellular physiology and well characterized protein synthesis inhibitors; however, mechanistic details, at the molecular level, have lagged behind. Currently, many of the missing details are being provided by high-throughput experiments that analyze the changes in the proteome or transcriptome in response to synaptic stimuli. As techniques are refined and technology improves in the coming decade we foresee the frequent application of high-throughput technology to provide both novel candidates and coordinated local expression of proteins that make individual synapses plastic and unique.

## Author Contributions

SN and KR-G designed and conceived the idea for this review. SN and KR-G reviewed the literature. SN made all figures and tables. SN wrote the first draft of the manuscript. KR-G edited the manuscript.

## Conflict of Interest Statement

The authors declare that the research was conducted in the absence of any commercial or financial relationships that could be construed as a potential conflict of interest.

## Footnotes

1 http://scotty.genetics.utah.edu

2 http://seqanswers.com

3 https://www.biostars.org

4 http://stackexchange.com

5 https://www.bioconductor.org

6 http://hannonlab.cshl.edu/fastx_toolkit

7 http://www.novocraft.com

8 http://genome.tugraz.at/genesisclient/genesisclient_faq.shtml
